# Health-Promoting Role of *Lactiplantibacillus plantarum* Isolated from Fermented Foods

**DOI:** 10.3390/microorganisms9020349

**Published:** 2021-02-10

**Authors:** Natalia Garcia-Gonzalez, Natalia Battista, Roberta Prete, Aldo Corsetti

**Affiliations:** Faculty of Bioscience and Technology for Food, Agriculture and Environment, 64100 Teramo, Italy; ngarciagonzalez@unite.it (N.G.-G.); nbattista@unite.it (N.B.); acorsetti@unite.it (A.C.)

**Keywords:** fermented foods, health benefits, *Lactiplantibacillus plantarum*, probiotics

## Abstract

Fermentation processes have been used for centuries for food production and preservation. Besides the contribution of fermentation to food quality, recently, scientific interest in the beneficial nature of fermented foods as a reservoir of probiotic candidates is increasing. Fermented food microbes are gaining attention for their health-promoting potential and for being genetically related to human probiotic bacteria. Among them, *Lactiplantibacillus* (*Lpb*.) *plantarum* strains, with a long history in the food industry as starter cultures in the production of a wide variety of fermented foods, are being investigated for their beneficial properties which are similar to those of probiotic strains, and they are also applied in clinical interventions. Food-associated *Lpb. plantarum* showed a good adaptation and adhesion ability in the gastro-intestinal tract and the potential to affect host health through various beneficial activities, e.g., antimicrobial, antioxidative, antigenotoxic, anti-inflammatory and immunomodulatory, in several in vitro and in vivo studies. This review provides an overview of fermented-associated *Lpb. plantarum* health benefits with evidence from clinical studies. Probiotic criteria that fermented-associated microbes need to fulfil are also reported.

## 1. Introduction

Traditional fermented foods are a rich reservoir of live and active microbes; indeed, they are considered the main source of lactic acid bacteria (LAB) in nature [1]. Besides their nutritional properties, fermented foods are garnering more attention for the microbes that they carry. These microbes are able to synthetize compounds during fermentation with high health-modulating potential, such as organic acids, short chain fatty acids, vitamins or peptides [2]. Beyond the ability to produce bioactive and nutritive compounds, food-associated microbes share other genetic and phenotypic traits similar to those present in probiotic strains of the same species [2]. Similar to probiotics, food-borne microorganisms can survive in the gastrointestinal (GI) tract and exert beneficial effects on the host. Although no empirical studies have provided precise numbers, it is estimated that large quantities of live LAB (approximately 10^8–^10^11^ CFU/d) are ingested through the consumption of fermented foods [3].

The consumption of fermented foods has been associated with numerous health benefits [2,4]. In recent years, there has been an increase in epidemiological and clinical reports that confirm their benefits, mainly associated with an improvement in health and a reduction in disease risk [5]. Recent investigations have pointed to a shaping of the gut microbiota when it is in contact with beneficial and safe microbes [3]. According to Marco and colleagues [3], the potential of food-borne microbes can be addressed in “the old friend hypothesis”, which suggests that “exposure to nonharmful or commensal microbes in foods may “engage” with the mucosal surfaces of the digestive tract, fine-tuning the immune system, bolstering gut function, and reinforcing the ability of the human symbiont to mitigate susceptibility to the development of chronic diseases”.

For decades, LAB have been extensively used in food fermentation due their nonharmful nature. Among LAB, one of the more versatile and promising species is *Lactobacillus plantarum* or, as it has recently been denominated, *Lactiplantibacillus* (*Lpb*.) *plantarum* subsp. *plantarum* [6]. *Lpb. plantarum* is a straight rod shaped (bacillus), Gram-positive, nonmotile, nonspore-forming, microaerophilic, mesophilic bacterium. Although is a catalase negative, some strains grown under special conditions possess true catalase and maganese-containing pseudocatalase activities [7]. The cell wall contains either ribitol- or glycerol- teichoic acid type, although some strains have an unusual teichoic acid. Peptidoglycan of the cell wall is of the meso-diaminopimelic acid (DAP) type. Included in the group of facultative heterofermentative bacilli, *Lpb. plantarum* strains possess cassettes of carbohydrate utilization genes that allow them to adapt to different ecological environments. Typically isolated from fermented foods, *Lpb. plantarum* strains can be encountered in a wide variety of niches, that includes the GI tract, stools, fermented foods, and plants, amongst others. For decades, *Lpb. plantarum* strains have been used in the food industry as starter cultures in the production of cheeses, olives and a wide variety of fermented foods and beverages, contributing to their organoleptic properties, flavor and texture [8]. One example food in which high concentrations of *Lpb. plantarum* can be found is table olives. Table olives are one of the oldest and most popular fermented foods, consumed all over the world and produced principally in the Mediterranean area (Italy, Spain and Greece); their main isolates, *Lpb. pentosus* and *Lpb. plantarum*, may be found in quantities of around 10^8^ CFU/g [9,10]. However, the impact of the consumption of these food-dominant strains on the host, either when consumed directly or as a part of a fermented food, is still unclear. Moreover, it is not yet known whether food-associated strains go on to become members of the gut microbiome. Currently, *Lpb. plantarum* strains are also being investigated for their health-promoting properties [7].

As will be discussed throughout the literature review, *Lactiplantibacillus plantarum* strains isolated from foods sources have been shown to display properties similar to those of therapeutic probiotic strains.

## 2. Selection Criteria for Health-Promoting Bacteria

So far, according to the FAO/WHO, only microorganisms isolated from the human GI tract are recommended for use as probiotics in humans [11]. Probiotics are live microorganisms that, when administered in adequate amounts, confer a health benefit on the host [12,13]. However, new evidence has highlighted the potential of food-associated microorganisms as probiotics [14]. For all strains, regardless of origin, the selection procedure follows the same criteria. Both food-related and commensal strains isolated from human GI tract have to be isolated, carefully characterized and demonstrated to provide a health benefit in order to be considered a probiotic. The FAO/WHO established a global standard for evaluating probiotics and health-promoting strains that can be summarized as follows [11,12]:

● **Strain identification**

According to the European Food Safety Authority (EFSA), an unequivocal taxonomic identification at strain level has to be performed for all microorganisms intentionally used in the food chain [15].

● **Safety properties**

Many lactobacilli have a long history of safe human use, having been used as starter cultures in fermented foods. As a result, many lactobacilli have been classified as “Generally Recognized as Safe” by the FDA, and have received “Qualified Presumption of Safety” by the EFSA. This notwithstanding, every strain intentionally used for industrial application or as a probiotic must be evaluated for safety with robust methods before it can be considered for real-life applications [16]. In 2019, the EFSA published a public consultation which stated the requirements for whole genome sequence analyses of microorganisms intentionally used in the food chain. The document “encourages” data to be obtained from whole genome sequencing (WGS) in order to perform accurate risk assessments. Data from in silico analyses can provide information about gene encoding for antimicrobial resistance, i.e., those related with virulence, pathogenicity and/or toxigenicity should be evaluated. 

● **Functional strain characterization for probiotic attributes**

Ability to tolerate acid/bile stress and adhesion to intestinal epithelial cells are the first properties to be evaluated. When consumed, bacteria must overcome several stresses encountered in the GI tract, including osmotic variations and low pH. Stresses to microorganisms which begin in the mouth, with the lysozyme contained in saliva, continue in the stomach, where the pH ranges between 1.5 and 3.0. Microorganisms can also be exposed to pepsin and lipase, and finally, in the upper intestine, to bile [17]. Thus, an important step toward the selection of potential probiotic candidates is to investigate strain behavior under conditions which mimic the GI tract, in particular, acid/bile tolerance. 

The lumen of the GI tract is composed of commensal microbiota, a mucus layer and epithelial cells. The monolayer of epithelial cells separates the intestinal mucosal, produced by goblet cells, and the commensal microbiota, from the immune cells, forming the gut epithelial barrier [18]. This intestinal epithelial barrier acts as a defense against infections, and its alterations have been associated with a number of disease states [19]. When consumed regularly, ingested bacteria or probiotics form part of the “transient microbiome”, i.e., they are not stable colonizers, but this transient passage allows them to interact with commensal bacteria and epithelial cells, and ultimately, to provide health benefits [20].

● **Clinical validation**

All probiotic candidates need validation of their health benefits through double-blind and randomized clinical studies in humans or in the organism for which they are intended [13]. 

## 3. Genomic Insight into Food-Borne *Lpb. plantarum* Species

Advances in next generation sequencing in recent years have led to the completion and publication of a significant number of *Lpb. plantarum* genome sequences. To date (December 2020), 560 *Lpb. plantarum* genomes are publicly available from the NCBI repository, of which 135 are complete. According to the published data, the genome size of *Lpb. plantarum* strains ranges from 2.91 to 3.7 Mbp in length, making *Lpb. plantarum* one of the largest genomes within the lactobacilli group, with a GC content of approximately 44%. Moreover, the number of coding sequences (CDSs) ranges between 1964 for *Lpb. plantarum* WHE92 to 3526 for *Lpb. plantarum* SRCM101258.

The first *Lpb. plantarum* to be completely sequenced was the strain WCFS1, isolated from human saliva, in 2003 [21]. However, it was not until 2009 that the first strain isolated from fermented foods was sequenced, the type strain ATCC14917^T^. Since then, a number of new genomes of *Lpb. plantarum* isolated from different sources have been sequenced and are available from the NCBI database (https://www.ncbi.nlm.nih.gov/genome/genomes/1108, accessed on 9 Febraury 2021). These strains encompass a wide variety of niches including but not limited to dairy products, meat products, vegetables and traditional fermented foods (i.e., kimchi) amongst others.

In-depth analysis of the genomic sequence of WCFS1 has deepened our understanding of the species and has served as a reference for further in silico studies based on its prediction/annotation of genes as a first approach to predict phenotypes. Major advances in the identification of genes related to GI survival, interactions with other microorganisms and the host, the ability to resist oxidative stress and the environmental adaptability of strains are described for *Lpb. plantarum* isolated from fermented foods. In this regard, *Lpb. plantarum* sequences encoding genes for adhesion to intestinal cells and mucus, such as mannose-specific adhesion (*msa*) and collagen binding proteins (cnaB), are both involved in the bacterial colonization and competition against pathogenic bacteria [22]. Food-borne *Lpb. plantarum* strains encodes genes for a number of stress-related proteins. The presence of the osmoregulatory system OpuC, the chaperones *gro*ES-*gro*EL and the *hcr*A-*dna*K-*dna*J-*Grp*E operon, NADH oxidases and peroxidases or thiol and manganese transporters confer advantages upon the strains, allowing them to survive in the harsh conditions of the GI tract [23,24]. Moreover, the presence in the genome of *Lpb. plantarum* strains of prophages and the CRISPR-Cas system are also considered advantageous, since both are involved in the defense against bacteriophage infections. A genome sequence analysis of *Lpb. plantarum* prophages indicated that Sha1 and Phig1 occur most abundantly [22]. Regarding the presence of the CRISPR-Cas system, most *Lpb. plantarum* display the class 2 CRISPR-Cas system (type II) with four genes, i.e., *cas*9, *cas*1, *cas*2, and *csn*2 [25].

It has been proposed that *Lpb. plantarum* strains possess in their genome a lifestyle adaptation region or lifestyle island, i.e., a region specific to *Lpb. plantarum* strains, mainly consisting of sugar transport and utilization, as well as serving an extracellular function, the encoding of genes [23]. This region appears to be key to the successful environment-adaptability of *Lpb. plantarum* strains. The capacity of *Lpb. plantarum* strains to ferment a variety of sugars has received significant attention, as their efficient transport systems lead to their high adaptability and their ability to survive in different ecological niches. Comparative genomic studies of *Lpb. plantarum* strains isolated from different sources showed that most of the genes encoded in the “lifestyle adaptation region” were nonconserved among strains, and encoded predicted plantaricin and exopolysaccharide biosynthesis genes, prophages and mobile elements [23]. These findings support the high genome plasticity of *Lpb. plantarum*, which, together with efficient metabolism, make them one of the most nomadic and versatile species.

In the following subsections, we will discuss major findings in exopolysaccharides and plantaricin production discovered in food-associated *Lpb. plantarum* strains.

### 3.1. Production of Exopolysaccharides

Exopolysaccharides (EPS) are high molecular weight and biodegradable polymers formed by monosaccharide residues of sugar and sugar derivatives and produced by a wide range of bacteria [26]. EPS can be subdivided based on their structure into two groups: hetero and homopolysaccharides, i.e., comprised of a repeating oligosaccharide, or a repeating monosaccharide, respectively.

EPS producing strains are typically described as “ropy” or “nonropy”, which describes the threads drawn with a needle from the surface of the colonies or fermented liquid containing the EPS producing culture [27]. EPS produced by LAB are secreted polysaccharides which can remain attached to the cell envelope in the form of a capsular polysaccharide (CPS), or be released into the surrounding environment [28]. The production of EPS by LAB is a widespread phenomenon which has received substantial attention in recent years based on attributes such as their biodegradability, biocompatibility and nontoxicity. In bacteria, EPS also has a protective nature; it allows bacteria to adhere to and recognize other bacteria and surfaces, and offers protection from heavy metals, phage infection and biofilm formation [29].

Genomic studies on *Lpb. plantarum* have highlighted the diversity in the genetic characterization and organization of the EPS loci within the species. Unlike other species such as *Lactobacillus (Lb.) johnsonii* and *Lb. helveticus*, which encode a single cluster, *Lpb. plantarum* harbors multiple EPS associated clusters, with up to five independent loci in an individual strain [30]. One of the best characterized EPS-clusters in *Lpb. plantarum* is that of strain WCFS1 [24]. The genome of WCFS1 encodes four chromosomal clusters of EPS genes, two involved in capsular polysaccharide formation (*cps*2A-J and *cps*4A-J) and another two clusters predicted to lack genes encoding chain-length control functions and a priming glycosyl-transferase (*cps*1A-I and *cps*3A-J) [31]. EPS producing *Lpb. plantarum* strains have been isolated from different sources, and their molecular characteristics are usually strain-dependent [31,32]. The strain *Lpb. plantarum* LP90, isolated from wine, possess *cps*3 and *cps*4 and a strain dependent *cps*2, while ST-III and ZJ316 encode the clusters *cps*3 and *cps*4 [33,34], and JDM1, P8 and 16 only encode the *cps*4 cluster [34]. Variability amongst EPS clusters in *Lpb. plantarum* strains is observed within clusters *cps*1A-I to *cps*3A-J. The gene cluster *cps*4A-J is the most conserved amongst the species [31]. Among the essential genes found within cluster *cps*4A-J are tyrosine kinases, phosphotyrosine phosphatase, a priming glycosyltransferase, glycosyltransferases, a flipase and a polysaccharide polymerase [30].

It has previously been shown that, in species with multiple EPS clusters like *Lpb. plantarum*, each cluster has a different function and a different biological impact. The study conducted by Remus and colleagues evaluated the four CPS gene clusters encoded by *Lpb. plantarum* WCFS1 and their impact in host-microbe interactions [31]. While deletions in *cps*1A-I did not affect to the production of polysaccharides, mutations in the other three clusters were shown to considerably reduce the levels of surface polysaccharides. However, only mutations in the *cps*1A-I cluster affected the molar mass and the composition of the EPS. Moreover, mutations in these clusters also impact on the toll-like receptor (TLR) recognition, and thus, on the activation of the Nuclear Factor kappa-light-chain-enhancer of activated B cells (NF-kB). When compared with the wild-type, individual mutations in the clusters appeared to slightly modify the TLR2-signaling response, while the deletion of all clusters elicited a drastically increased NF-kB activation [31].

The production of EPS has had a significant impact on the pharmacological and food industries due its physicochemical properties. It has been shown that EPS produced by some LAB improves food texture, affecting the rheological properties, such as mouthfeel and matrix formation, along with the finished quality of fermented foods [35]. Moreover, beneficial effects such as anticarcinogenicity, antithrombotic, antioxidant and immunomodulating activities have also been attributed to EPS [36]. EPS isolated from the food-associated strains C70, Y0175 and OF101, isolated from Chinese Paocai, and a traditional fermented cereal beverage, respectively, showed antioxidant properties [37,38]. In addition, EPS isolated from camel milk, KX041 showed both immune activity and DPPH/ABTS radical scavenging activities [39]. Antitumor and antibacterial properties have been also observed from the EPS isolated from *Lpb. plantarum* strains MTCC9510 and 86, respectively [40,41]. For these reasons, the scale-up of EPS production has been studied, as well as their applicability in food and pharmaceutical industries [35,36].

### 3.2. Production of Bacteriocins

Bacteriocins are, by definition, ribosomally synthesized peptides used by bacteria as a defense mechanism against other bacteria. Most of the bacteriocins produced by LAB are small, cationic, heat-stable, amphiphilic and membrane-permeabilizing peptides [42]. Bacteriocins produced by *Lpb. plantarum* are known as plantaricins. In recent years, bacteriocins produced by LAB have gained interest in industry due to their potential role as biopreservatives [43]. Since they can be degraded by proteolytic enzymes, bacteriocins are presented as a natural, safe and effective strategy to combat foodborne pathogens and spoilage bacteria in comparison with current chemical preservatives or the use of antibiotics [44]. However, the use of bacteriocins has some limitations, such as the efficacy of pathogen elimination and their elevated cost.

Characterization and complete understanding of the bacteriocin loci is important, since it has been proven that variations in gene sequences, composition and organization may affect the antimicrobial activity of bacteriocins [45]. There are six main features of plantaricins producing by *Lpb. plantarum* strains [46]. All plantaricins are produced as precursors with a double glycine moiety by the genes *pln*E and *pln*F, and further exported by the PlnG and PlnH proteins [47]. Bacteriocins are divided into four categories, based on structure, molecular weight, heat persistence and molecular organization [48]. The majority of the plantaricins produced *by Lpb. plantarum* are usually included in both class I and II. Class I includes bacteriocins which are post-translationally modified, containing a lanthionine, and are commonly named lantibiotics. In this group, plantaricins C and W are found [49,50]. In general, bacteriocins belonging to class II are heat-stable, unmodified and nonlanthionine-containing. Class II is a heterogeneous group of bacteriocins subdivided into class IIa, pediocin PA-1 like bacteriocins; IIb, two-peptide bacteriocins; IIc, circular peptide bacteriocins; and IId, linear and single-peptide bacteriocins without a pediocin-domain. Plantaricins JK, EF S and NC8 belong to class IIb, while plantaricin STSH8, C19 and 423 belong to class IIa [16,51,52,53]. Production of plantaricins JK and EF is induced by plantaricin A, belonging to class IIc [54]. Finally, class III consists of large heat labile bacteriocins poorly represented in LAB. *Lpb. plantarum* strains, producing one or more types of plantaricins, have been isolated from different fermented foods [16] (Table 1). Generally, *Lpb. plantarum* species are considered a source of a variety of strong plantaricin producers.

## 4. Health Benefits of Food-Associated *Lpb. plantarum* Strains

**Figure 1:**
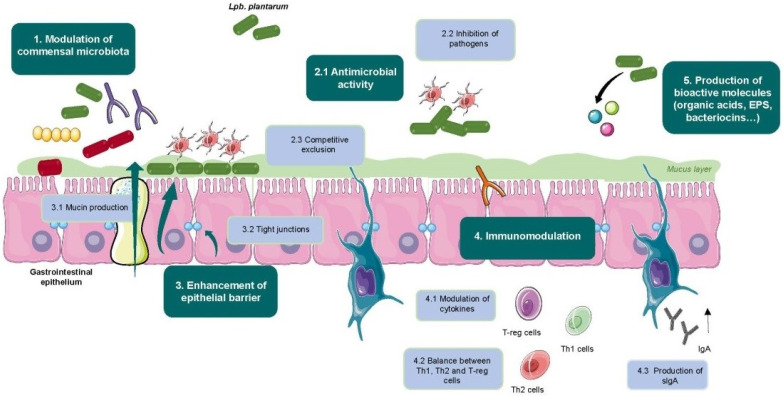
Mechanisms of action of *Lpb. plantarum* health benefits. Graphical illustrations were created using items from Servier Medical Art by Servier, available at https://smart.servier.com/ under a Creative Commons Attribution 3.0 Unported License.

In the following subsections, we will describe some of the most characterized health-beneficial activities of *Lpb. plantarum* strains isolated from fermented foods. In general, the mechanisms by which probiotic bacteria mediate their health benefits are: (1) modulation of commensal microbiota; (2) exclusion or inhibition of pathogens; (3) enhancement of the intestinal epithelial barrier by increasing mucin production and tight junctions formation; (4) modulation of the immune system; and (5) production of bioactive molecules. Figure 1 presents a simplified graphic of the main mechanisms of action of *Lpb. plantarum* strains which will be further described in this review.

### 4.1. Antimicrobial Activity

Among the beneficial effects of *Lpb. plantarum*, one of the most studied and desirable among probiotic properties is their antimicrobial potential. It has been shown that *Lpb. plantarum* species are endowed with a wide spectrum of antibacterial activity against many food spoilage microbes (such as bacteria, yeasts and molds) and various enteropathogenic bacteria [86]. Therefore, several *Lpb. plantarum* strains have been considered as promising probiotic candidates to be applied in the food industry and human medicine as bio-preservatives and bio-therapeutics alternatives, respectively. Recent studies showed the ability of food-associated *Lpb. plantarum* strains to inhibit both Gram-positive and -negative bacteria, such as *Listeria monocytogenes*, *Staphylococcus aureus, Enterococcus*, *Bacillus, Clostridium*, *Pseudomonas aeruginosa*, *Helicobacter pylori*, *Yersinia enterocolitica*, *Campylobacter jejuni, Klebsiella*, *Salmonella*, *Shigella* and *Escherichia coli* (including *E. coli* 0157:H7) among others (Table 1). Good antifungal activity has also been shown against various yeast and mold species, including *Aspergillus*, *Candida* spp. and *Fusarium* [87]. Several studies have examined the antagonist effects of *Lpb. plantarum* strains isolated from different fermented foods against food spoilage and/or pathogenic microorganisms [88,89,90,91]. The antimicrobial activity is mainly exerted by the production of antimicrobial compounds, such as organic acids, hydrogen peroxide, EPS and bacteriocins production; this, as well as many others beneficial properties, should be considered strain specific, and thus, need to be characterized on a strain level.

Regarding the inhibition of food spoilage and food pathogen bacteria, *Lpb. plantarum* species have been applied as starter cultures in the fermentation processes of many fermented foods (sauerkraut, table olives, dairy products, fermented sausages, etc.). Such fermentation process improve both food quality and safety, and prolong the shelf-life of final products by inhibiting food spoilage microbes, mainly through organic acid production and competition for nutrients [92]. As olives ferment, the lactic acid production by *Lpb. plantarum* lowers the pH, inhibiting the growth of spoilage microbes which are sensitive to acidic conditions, significantly improving microbiological stability and food safety [10]. Strong inhibitory activity has been reported of lactic acid at low pH against Gram-negative bacteria (i.e., *Escherichia coli* and *Salmonella* Enteritidis), spore-forming bacteria and diverse yeasts and molds [93]. The antifungal activity of *Lpb. plantarum* is mainly due to the production of organic acids [94], 3-hydroxylated fatty acids (i.e., 5-oxododecanoic acid, 3-hydroxy decanoic acid and 3-hydroxy-5-dodecenoic acid [95]) and cyclic dipeptides as cyclo (Gly-Leu), cyclo (Phe-Pro), cyclo(Phe-OH-Pro), cyclo (Leu-Pro) [96].

Besides the above-mentioned antimicrobial compounds, *Lpb. plantarum* strains are also producers of EPS and bacteriocins, leading to remarkable inhibition of the activity of pathogens [87]. Currently, bacteriocins are presented as natural, safe and effective strategies to outcompete food-borne pathogens and spoilage bacteria in comparison with current chemical preservatives or the use of antibiotics [44]. The spectrum of action of plantaricins is extremely diverse. Normally, most plantaricins are active against either Gram-positive or -negative bacteria, although there are some cases in which plantaricins are active against both (such as plantaricin ZJ5 or LP84) [16] (see Table 1). The potential of some plantaricins, such as plantaricin C11 and NA, is remarkable; these inhibit *Listeria monocytogenes*, an invasive foodborne pathogen [53]. Table 1 reports the most characterized plantaricins produced by *Lpb. plantarum* strains isolated from various fermented foods. Regarding EPS production, the bioactivity of these compounds produced by *Lpb. plantarum* against pathogens, such as antiadhesion and antibiofilm properties, has been described in some studies [29,36]. Pathogen persistence and biofilm production, due to the resistance of some pathogens to antibiotics, can lead to chronic infections and present serious challenges in the food industry [97]. The antimicrobial activity of *Lpb. plantarum* has gained interest in the food industry due to their potential role as biopreservative agents [43].

On the other hand, *Lpb. plantarum* have been shown to be able to inhibit a wide spectrum of host-pathogenic bacteria, including the most harmful bacteria, *Staphylococcus aureus* and *Escherichia coli*; as such, they are considered a promising antibiotic alternative [98]. Zhang and coworkers reported significant pathogen inhibition by *Lpb. plantarum* ZDY 2013, a strain isolated from fermented bean. *Lpb. plantarum* ZDY 2013 was shown to outcompete and inhibit strains of *Bacillus cereus*, well-known enterotoxic and pathogen species, as well as to be effective as a pretreatment for the prevention of *Helyicobacter pylori* infection and related gastric mucosal inflammation [99].

*Lpb. plantarum* species have been also investigated for potential antimicrobial properties toward human skin pathogens, e.g., *Pseudomonas aeruginosa* and the methicillin-resistant *Staphylococcus aureus* species, in order to potentially use some strains as bio-control agents for wound infections [100]. Probiotic lactobacilli have been widely investigated as possible therapeutic alternatives for the prevention of recurrences of vulvovaginal candidiasis, a common infection by *Candida albicans* among women [101]. Additionally, some studies have reported strong in vitro and in vivo antifungal activity against *Candida albicans* by *Lpb. plantarum* strains [102,103]. Interestingly, Beck and coworkers recently investigated three *Lpb. plantarum* strains isolated from Kimchi, a Korean traditional fermented food, for their antimicrobial activities against *Candida albicans* and *Gardnerella vaginalis,* suggesting their potential as probiotic candidates for the treatment of mucosal infections [103].

Based on all the evidence confirming the potent antimicrobial activity of *Lpb. plantarum* against a wide variety of food-spoilage and pathogenic microbes, as well as their well-established application as starter cultures, food-associated *Lpb. plantarum* strains are promising as bio-preservative agents in the food industry and/or as probiotics for alternative biotherapies in medicine.

### 4.2. Antigenotoxic and Antimutagenic Activity

Daily exposure to a huge variety of environmental and food-related mutagens, mainly linked to Western diets and modern lifestyle, has led to increased scientific interest in dietary interventions to modulate the risk of genotoxicity and related GI disease. In the gut, a variety of genotoxic compounds (mainly delivered by foods) can often be found. They can be broadly defined as primary food mutagens (i.e., mycotoxins, vegetable glycosides), secondary food mutagens *(*i.e., originating from cooking processes such as polycyclic aromatic hydrocarbons and heterocyclic amines) and endogenous compounds (i.e., nitrosamines) [104]. It has been shown in preclinical and clinical studies that they are involved in the development of different tumors, such as colorectal, prostate and breast cancers [105,106] In addition, we are constantly exposed to a wide variety of environmental and exogenous compounds, commonly used in cosmetics, food packaging and/or in thousands of everyday products, such as heavy metals, phenolic derivates (i.e., BPA), phthalates, nitrosamines, Polybrominated Diphenyl Ethers (PBDEs), Polychlorinated Biphenyls (PCBs) and many others, also called endocrine disruptors (EDs). Recently, exposure to EDs has been linked to metabolic disorders, such as diabetes, obesity [107] and many other adverse outcomes, including carcinogenic effects through DNA damage in different organs (i.e., liver, pancreas and intestine) [108].

However, some microbial communities that inhabit the gut have antigenotoxic properties that can cause significant reduction in the biological activity of these chemical compounds [109]. These protective activities have also been reported for some fermented foods, such as fermented soy milk [110]. Epidemiological and clinical-experimental evidence also confirmed the intimate diet-health relationship, in which the commensal bacteria play a key role in the modulation of genotoxic and mutagenic risk at the intestinal level [111,112].

From this perspective, several studies have noted that food-associated microbes that are widely ingested may be related to reduced colon cancer incidence from environmental risk factors, such as dietary and exogenous xenobiotics [106]. Recently, Garcia-Gonzalez and coworkers extensively reviewed both in vitro and in vivo studies, providing supportive evidence that food-associated and/or probiotics LAB have the ability to play a protective role at the GI level by inhibiting the biological activity of genotoxic compounds, and thus preventing DNA damage, an early event in the carcinogenesis [108]. It has been suggested that food-associated LAB can reduce the genotoxicity of such chemical molecules by either binding or bio-converting them to unreactive compounds [113], although the exact mechanism for this is not yet fully understood. Among LAB, *Lpb. plantarum* strains isolated form Italian dairy products have been reported to be effective against the nitroarene, 4-nitroquinoline-1-oxide (4-NQO) and the alkylating agent, N-methyl-N’-nitro-N-nitrosoguanidine (MNNG), two potent model genotoxins [104,109]. Moreover, Walia and coworkers, investigating the DNA-bioprotective activity of microbes associated with fermented foods of the North-Western Himalayas, reported a high genotoxicity inhibition against 4-NQO and furazolidone (>90%) of some *Lpb. plantarum* strains, statistically similar to that expressed by the well-known probiotic strain, *Lacticaseibacillus rhamnosus* strain LGG (88.9%). Prete and coworkers, carried out a screening within *Lpb. plantarum* species, assessing the antigenotoxicity of 18 *Lpb. plantarum* strains isolated from different fermented foods (table olives, sourdough and raw-milk cheeses) against 4-NQO. Their study confirmed the considerable potential of *Lpb. plantarum* species to inhibit the genotoxic effect of carcinogenic compounds, albeit with evident strain-specificity [114]. The food-associated *Lpb. plantarum* DNA-bioprotective effect has been also established on human colon adenocarcinoma (Caco-2) cells against Aflatoxin B1, one of the most well-known mycotoxins with hepato-carcinogenic effects [115].Recently, *Lpb. plantarum* LUHS135 and *Lacticaseibacillus* *paracasei* LUHS244 from fermented cereals were investigated as candidates for the reduction of some mycotoxins (i.e., aflatoxin B1, ochratoxin A, HT-2 toxin, T-2 toxin [116].

Beside their bioprotective role against food-related mutagens, *Lpb. plantarum* strains have been also investigated for their ability to counteract environmental mutagens, showing a bio- protective effect in the case of waterborne cadmium [117] and triclosan exposure [118]. The ability of food microbes and/or probiotics to detoxify and degrade environmental chemical compounds is now emerging as a new bioremediation tool. Recent studies reported potential in vitro ability of dairy LAB to bind BPA [119] and pesticides [120]. A recent study explored the antigenotoxic activity of two *Lpb. plantarum* probiotic strains, IMC510 and IMC513, previously characterized for other functional activities [114,121,122] against two different EDs [108], confirming the role of *Lpb. plantarum* in inhibiting genotoxicity and DNA damage. Considering the increasing need for bio-protection and bio-remediation from carcinogenic and mutagenic compounds, the ability of microbes to protect from DNA-damage is emerging as an innovative functional property, representing the basis for new, bio-protective diet interventions to reduce chronic gut pathologies, that deserve to be investigated with further in vivo studies.

### 4.3. Bile Salt Hydrolase Activity

Bile is one of the environmental challenges that microorganisms must endure in order to survive in the gastrointestinal environment. Typically, a liter of bile is secreted by the liver into the intestinal tract every day, which represents a serious challenge for ingested strains [123]. Bile is a digestive secretion that is required for the emulsification and intestinal absorption of dietary fats, lipids and lipophilic vitamins. Bile acids (BAs), cholesterol, phospholipids and conjugated bilirubin are among the major constituents of bile. BAs are hydroxylated steroids synthesized in the liver from cholesterol, stored in the gall bladder, and released in the small intestine following food consumption. They play a major role in the emulsification and solubilization of lipids, facilitating their absorption and digestion, and in the elimination of cholesterol [123]. Bile acids are surface active, amphipathic molecules, and their ability to act as detergents also allows them to interact with bacterial membrane lipids, causing cell membrane disruption as well as triggering DNA damage, thereby conferring potent antimicrobial properties on bile [124]. Prior to secretion in bile, primary BAs are conjugated at their side chains with either taurine (tauro-conjugated) or glycine (glyco-conjugated). After being released into the duodenum, conjugated bile acids are subject to chemical modifications by the gut microbiota through bacterial bile salt hydrolase (BSH) enzymes [123]. Once they are in their deconjugated form, after removal of glycine or taurine, bile salts can be excreted with the feces due to their lower water solubility [125]. In this respect, the ability to hydrolyze bile salts, also known as BSH activity, has been included among the criteria for probiotic strain selection [126]. In terms of bacterial survival in the gut environment, it is generally considered necessary to evaluate the ability of potentially probiotic bacteria to endure bile acid-related stress [126]. The production of BSH enzymes provides bacteria with a mechanism with which to survive in the gastrointestinal tract, as conjugated bile acids are known to be toxic to bacteria, contributing to both microbial bile resistance and colonization of the GI environment. The ability to metabolize bile acids, which is a conserved microbial adaptation, is considered a common feature of gut microorganisms and is distributed across the major phyla of bacteria in the gut, as well as the gut Archaea [127]. BSH deconjugation activity has been primarily characterized among GI commensal species such as *Bifidobacterium* [128], *Clostridium* [129], *Enterococcu*s [130], *Listeria* [131], *Lactobacillus* [132,133], including *Lpb. plantarum* species [134]. *Lpb. plantarum* WCFS1 was the first *Lpb. plantarum* strain in which *bsh* genes were described [135]. Functional analyses performed on other *Lpb. plantarum* strains revealed a family of four bile salt hydrolase proteins [125], among which BSH1 seems to be the main one responsible for the ability of *Lpb. plantarum* to metabolize BAs. Recently, Prete and colleagues reported early evidence of BSH activity in food-associated *Lpb. plantarum* strains, even though they were not gut-associated strains [136]. Moreover, variations in BAs deconjugation were found among the strains, which confirmed the strain-dependent nature of this property, that cannot be generalized within a species or a genus, as previously reported by several investigators [123]. 

Currently, microbial bile tolerance is increasingly gaining attention due to its potential impact on physiological processes; thus, BSH activity could be a desirable feature in strain selection. In this respect, microbial BSH activity was recently identified as a form of gut microbial activity that mediates a microbe-host dialogue that functionally regulates host lipid metabolism and plays a crucial role in cholesterol metabolism. Bile acids act as biological signaling molecules, whose interactions with some host receptors such as the nuclear bile acid receptor (also known as farnesoid X receptor, FXR) or the bile acid-activated membrane G protein-coupled receptors, TGR5 (aka Gpbar-1, G-protein-coupled bile acid receptor) appear to play a role in stimulating energy metabolism, protecting liver and intestine from inflammation and steatosis, and improving insulin sensitivity, as well as playing a significant role in weight loss [137,138]. In particular, FXR is involved in regulating BA synthesis and enterohepatic circulation in the liver and intestine, in which FXR activation leads to lower BA and cholesterol levels by reducing BAs synthesis through the inhibition of hepatic cholesterol 7α-hydroxylase (CYP7A1) and sterol 12α-hydroxylase (CYP8B1), and by enhancing cholesterol excretion through the activation of bile-salt transporter such as bile-salt export pump (BSEP), thereby playing a regulatory role linked to anti-inflammatory and metabolic benefits [139]. It has been shown that the administration of probiotic bacteria can be a preventive strategy to modulate cholesterol serum levels and related cardiovascular diseases. Several in vivo studies have confirmed that the reduction of cholesterol and triglyceride levels in animal models is mainly associated with the presence of microbial BSH ability [138,140,141]; this has also been confirmed by clinical trials which sought to evaluate the impact of BSH-active probiotic on cholesterol metabolism [142]. Among their biological roles, BAs act as signaling molecules in glucose homeostasis and energy expenditure via activation of TGR5 receptor that stimulate the browning of white adipose tissue, postprandial thermogenesis improving whole-body glucose and lipid metabolism [137,143]. It has also been reported that the activation of TGR5 is involved in innate immune responses [143]. A bile acid-adaptive immunity axis has been demonstrated, in which activation of the vitamin D receptor (VDR) by the secondary bile acid, lithocholic acid (LCA), directly mitigates the Th cell inflammation, thereby modulating the adaptive immune response, which is fundamental in all inflammatory conditions [143]. Besides immunity, the VDR receptor is involved in the regulation of many others biological functions such as cellular proliferation and differentiation, calcium homeostasis and xenobiotic detoxification [139]. Recently, it has been shown that unconjugated BAs can influence the regulation of host circadian gene expression, acting as microbial-derived regulators of circadian rhythm, whose alteration is known to be associated with obesity and metabolic dysfunctions [144]. Overall, the multiple biological roles of BA molecules reflect the intimate host–microbe crosstalk. Considering that fermented foods are rich in microbes that, once ingested, can actively contribute to host metabolism and homeostasis, microbial ability to modulate the profiles of BAs shows great promise in terms of food strategies to improve human health.

### 4.4. Antioxidant Properties

Nowadays, there is much interest in the effects of reactive oxygen species (ROS) and related oxidative stress that cause many alterations and inflammatory conditions in the gut. Diet, carrying both food and microbial components, is primarily responsible for the production of pro-oxidant and ROS precursor molecules in the gut environment. Recently, dietary interventions using bioactive antioxidants such as food extract or probiotic strains have been investigated as an innovative natural approach to treat oxidative stress disorders and related chronic and inflammatory diseases [145]. ROS such as hydrogen peroxide (H_2_O_2_), hydroxyl radical (•OH), and superoxide anion (•O2¯) are produced during cellular metabolism and are key factors in important processes such as cell signaling, ion transportation and gene expression [146]. However, ROS accumulation could lead to the oxidative injury of biomolecules including lipids and proteins. This oxidation damage is known to cause multiple associated diseases, so balance and maintenance of redox homeostasis are essential for maintaining correct cell functions. In particular, food-associated *Lpb. plantarum* strains have been widely investigated due to their antioxidant properties against ROS and free radicals. The mechanisms underlying the antioxidant activities of food-borne *Lpb. plantarum*, and other probiotic strains appear to be multifactorial. The production of antioxidant metabolites (such as folate, butyrate or glutathione), upregulation of antioxidant host genes (such as superoxide dismutase or catalase), downregulation of genes related with ROS production, or modulating intestinal microbiota are some of the existing mechanisms known in several probiotic strains. The antioxidant properties of *Lpb. plantarum* strains can be evaluated directly, against molecules and radicals, or quantified as oxidative stress in a cell model, like the dichlorofluorescein (DCF) assay [147].

Chemical assays to test the antioxidant activity of strains include 1,1-diphenyl-2-picrylhydrazyl (DPPH) radical scavenging, hydroxyl radical scavenging (HRS) method and reducing power, among others. It is always recommended that antioxidant activity be evaluated using more than one method, and that the results be confirmed with an in vitro assay. Although correlation between chemical assays, in vitro approaches and confirmation of the in vivo activity cannot always be shown, it is better to perform this first screening to verify the antioxidant activity of particular strains. Xing and collaborators showed, by assessing that the antioxidant activity of a collection of lactobacilli strains, that *Lpb. plantarum* CCFM8661 displayed weak antioxidant activity in chemical assays, but proved its efficacy in inhibiting the radical-mediated damage on HepG2 cells [148]. Moreover, a study carried out with a collection of *Lpb. plantarum* and *Lpb. paraplantarum* strains isolated from fermented foods (khalpi, gundruk, sinki and bamboo) showed antioxidant activity by DPPH assay [149]. *Lpb. plantarum* K46 was able to tolerate hydrogen peroxide and exhibited good free radical scavenging activity [150]. The same properties were found for the strain DM5, which also showed strong antioxidant ability against hydroxyl radicals, DPPH activity, hydrogen peroxide resistance and inhibition of ascorbate [151]. Not only did the cells show antioxidant properties, but so too did heat-kill bacteria and cell-free extracts. Cell-free extracts of three *Lpb. plantarum* strains (C88, C10 and K25) isolated from traditional Chinese fermented foods showed strong hydroxyl radical scavenging activity [146], and both the supernatant and cell homogenate of *Lpb. plantarum* MA2, isolated from Tibetan kefir, exhibited glutathione peroxidase activity and superoxide dismutase activity [152]. Even though *Lpb. plantarum* does not have as complex a regulation system to defend against oxidation as eukaryotic cells, the presence of some enzymes such as nicotinamide adenine dinucleotide (NADH)-oxidase, superoxide dismutase, NADH peroxide and nonheme catalases is crucial when oxidative stress occurs [153]. Other studies have identified the EPS produced by LAB as being responsible for the antioxidant capacities of *Lpb. plantarum* strains [154].

Understanding the molecular mechanism behind the antioxidative properties of *Lpb. plantarum* and how microbe–host interactions can ameliorate oxidative inflammation is still an open challenge for researchers. Besides the direct neutralization of ROS, it has been shown that microbial cells can modulate or even block inflammatory pathways via modulation of ROS levels [155]. Recently, some *Lpb. plantarum* probiotic candidates, isolated from different fermented foods, were investigated for their antioxidant potential with a combined approach of in vitro chemical and cell-based assays [122]. Prete and coworkers found a potential dualistic effect of *Lpb. plantarum* in an intestinal cell model upon oxidative stress. In particular, their results suggested a preventive or protective effect of food-associated *Lpb. plantarum* strains based on the physiological status of the intestinal mucosa, i.e., either healthy or inflamed, suggesting an intimate and complex microbe–host interaction that goes beyond direct ROS neutralization [122]. Recent studies have noted that both commensal and probiotic microbes are directly associated with intestinal signaling via ROS modulation, and can influence different transduction pathways involved in restoring epithelial barrier function and gut inflammation [156,157], thereby providing evidence for the therapeutic use of food-associated *Lpb. plantarum* to ameliorate GI disorders related to oxidative and inflammatory stress.

### 4.5. Immune Modulation 

One of the most attractive properties of commensal bacteria and probiotics is their contribution to host homeostasis by modulating the immune system. This modulation is driven by the production of immunoregulatory compounds and/or by direct stimulation of immune and epithelial cells [158]. Although the colonization of probiotics in the GI tract is transient, during their passage, bacteria are able to interact with both commensal microbes and epithelial cells. This brief contact allows probiotics and/or ingested bacteria to modulate the activity of epithelial cells, which, in turn, may activate immune cells, such as dendritic cells and macrophages [159]. Diverse effects have been associated with probiotics: increased secretory immunoglobulin A (sIgA) production, regulation of pro- and anti-inflammatory cytokines production, and modulation of the balance between T-helper (Th1, Th2) and regulatory T-cells (T-regs) [160].

Host intestinal and immune cells recognize commensal and foodborne bacteria through pattern recognition receptors (PRRs). In particular, the main receptors involved in the host–microbe crosstalk are TLRs and leucine-rich repeat containing receptors (NLRs). TLRs and NLRs recognizing microbial components are responsible for initiating the immune response [161]. TLRs are transmembrane receptors which can either be expressed on the cell surface or on intracellular organelles of immune and nonimmune mammalian cells, such as dendritic cells, natural killer cells, epithelial and endothelial cells, amongst others [162]. TLRs can recognize different components of the bacterial cell wall such as lipoteichoic acid (LTA) and lipopolysaccharides (LPS). In contrast, NOD1 and NOD2 (within the NLR family) act as cytoplasmic microbial sensors of intracellular bacteria, recognizing peptidoglycan [163].

Commensal and probiotic bacteria share the ability to interact with intestinal and immune cells, inducing the production of selected cytokines; food-borne *Lpb. plantarum* strains have been shown to possess the same ability. *Lpb. plantarum* strain 06CC2 isolated from Mongolian dairy products can increase the release of interleukin (IL)-12 in coculture with murine macrophages J7741.A. Moreover, the oral administration of the bacteria was shown to induce Th1 cytokine production, activating the immune response in normal mice [164]. In addition, pretreatment with some *Lpb. plantarum* strains isolated from different sources showed the ability to reduce IL-8 concentrations in inflamed colonic cells (NCM460) [121], as well as to modulate the IL-23/IL-17 axis [122]. *Lpb. plantarum* LC27, isolated from kimchi, was able to offset the ethanol-induced effects in macrophages, KATO III cells and mice by inhibiting the activation of NF-kB and the consequent release of IL-8 [165]. Vitali and colleagues determined the probiotic potential of several autochthonous lactic acid bacteria, in which *Lpb. plantarum* strains were encountered [166]. An immunomodulation assay showed that food-associated *Lpb. plantarum* strains were able to induce the release of cytokines in peripheral blood mononuclear cells (PMBC). In particular, *Lpb. plantarum* POM42 was able to stimulate the largest number of cytokines with anti-inflammatory activity (IL-4, IL-1ra, IL-10 and IL-13) [166]. Preincubation of HT-29 cells with *Lpb. plantarum* strains FRP16, isolated from dairy products, was able to inhibit the production of IL-8 induced by *Salmonella Typhimurium* DT104 [167]. The capacity of *Lpb. plantarum* strains to modulate cytokine release was also observed when bacteria were heat-killed. Heat-killed *Lpb. plantarum* 137 isolated from a typical component of the Filipino diet induced the production of IL-12 and Interferon- γ (IFN-γ) by spleen cells in vitro [168].

In mice, treatment with *Lpb. plantarum* JLK0142, isolated from fermented dairy tofu was shown to increase the intestinal sIgA and the serum levels of IL-12 and Tumor necrosis factor (TNF)-α cytokines [169]. The same trend was observed in six-week-old BALB/c mice fed with *Lpb. plantarum* strains isolated from cheeses [170]. Consumption of *Lpb. plantarum* strains increased the phagocytic activity of peritoneal macrophages and the number of IgA-producing cells. In addition, a protective immune response was related with the consumption of *Lpb. plantarum* YU, isolated from traditional Japanese fermented foods [171]. As reported for other food-borne *Lpb. plantarum* strains, consumption of *Lpb. plantarum* YU increased IL-12 release and IgA activity, leading to enhancement of the Th1 immune response. Probiotic consumption has been linked with an increase of sIgA, one of the components of the humoral adaptative immune response. Production of IgA and further translocation to the intestinal lamina propria enhance the epithelial barrier by immune exclusion of pathogens [160]. Contact between bacteria and epithelial cells seems to be mediated by PRRs, in particular, by TLRs. As mentioned, TLRs are transmembrane receptors that respond to microbial surface-associated MAMPs. Once TLRs recognize bacteria, they are able to transduce this signal by recruitment of myeloid differentiation primary response 88 (Myd88), which, in turn, induces the Myd88-dependent signaling pathway for NF-kB and Mitogen-activated protein kinase (MAPK) activation [158]. The critical role of TLRs in the induction of immune responses has been proven. The *Lpb. plantarum* strain isolated from kimchi, CLP-0611, was shown to be capable of inhibiting IL-1β and IL-6 expression, as well as NF-KB and Activator protein-1 (AP1) activation in LPS-macrophages, and of inhibiting NF-kB activation in 2,4,6-trinitrobenzne sulfonic acid (TNBS)-induced colitis mice. Both results may suggest that the effects of *Lpb. plantarum* in modulating immune response is mediated by the regulation of the canonical TLR/NF-kB signaling pathway [172]. A study carried out by Ren and colleagues [173] showed that the stimulation of THP-1 by a collection of several food-associated *Lpb. plantarum* strains was TLR-dependent. 

The effects on the immune system associated with food-borne *Lpb. plantarum* strains are diverse. Different biological responses after TLR-activation may be due to small differences in the composition and structural organization of the cell wall of the bacteria, along with EPS production [174]. What seems clear is that the immunoregulatory effects on gut homeostasis from probiotics or ingested bacteria are not the result of a single activation of a PRR, but rather, of a synergistic combination of TLR and NLR activation. Upregulation of TLRs by probiotics or commensal bacteria could be considered as a defense mechanism, because it has the potential to keep the immune system on alert. Increment of sIgA production, regulation of cytokine production and modulation of the balance between Th1, Th2 and regulatory T-cells seem to be the key factors in the mechanism of action of probiotics.

## 5. Clinical Studies

The health benefits of probiotics have to be demonstrated in at least one successful human trial supporting the health claim for which probiotic strains would be dispensed [13]. Following these criteria, a substantial body of evidence confirmed the successful use of diverse human probiotic *Lpb. plantarum* strains as a dietary intervention to prevent and/or ameliorate some widespread diseases, especially acute and chronic GI infections (i.e., *C. difficile* and *H. pylori* infections) [99,175], gut inflammatory syndrome (i.e., Irritable Bowel Disease (IBD) and Ulcerative Colitis) [176,177] cardiovascular diseases [178], hypercholesterolaemia and obesity [179,180], diabetes [181], gynaecological diseases [182] as well as colon cancer [183] and cognitive impairments [184].

Recently, *Lpb. plantarum* strains, isolated from a variety of fermented foods, have been applied in clinical studies as dietary interventions in both healthy and diseased subjects (Table 2). It is worth noting that the majority of *Lpb. plantarum* strains were isolated from ethnic traditional fermented foods, (such as kimchi, Taiwan mustard greens, Mongolian sour milk and *dadih*, an Indonesian traditional, spontaneously fermented buffalo milk), confirming the fundamental role of fermented foods in health promotion; the consumption of such foods in Western societies is, nowadays, is nearly lost. 

In particular, food-associated probiotic strains have been administrated either alone, as capsules or powder (i.e., *Lpb. plantarum* PS128, P8, DR7) or through probiotic formulations in combination with other probiotic strains belonging to different species (i.e., *Latilactobacillus (Lat.)* *curvatus* HY7601 and *Lpb. plantarum* KY1032, *Lpb. plantarum* UBLP-40 in UB0316 multispecies formulation), as well as within fermented food diets to synergistically enhance the beneficial effects (i.e., *Lpb. plantarum* C29 in fermented soybean powder, *Lpb. plantarum* YIT 0132 in fermented citrus juice). Various *Lpb. plantarum* strains have been successfully administrated in overweight subjects with hypertriglyceridaemia and body adiposity to investigate their triglyceride-lowering effects. Interestingly, a 12-week administration of two strains, *Lat. curvatus* HY7601 and *Lpb. plantarum* KY1032 (isolated from Korean traditional fermented cabbage), showed significant triglyceride-lowering effects through reductions in plasma metabolites, fatty acid primary amides and lysophosphatidyl choline (lysoPC) [185], and a subsequent increase in apolipoprotein A-V and LDL cholesterol [186] in overweight but nondiabetics adults (n = 92 and n = 128 respectively). In addition, evidence has been presented of body weight loss and reduction in adiposity after administration of *Lat. curvatus* HY7601 and *Lpb. plantarum* KY1032 in two human trials involving overweight nondiabetic adult patients with hypertriglyceridaemia [187,188]. Costabile and coworkers found a similar beneficial outcome of food-associated *Lpb. plantarum* ECGC 13110402, a strain selected for its notable BSH activity, in lowering cholesterol levels in a clinical study enrolling normal to mildly hypercholesterolaemic participants [189]. In line with the potential probiotic impact on metabolic syndrome, multistrain probiotic formulation UB0316, containing *Lpb. plantarum* UBLP-40, was recently applied as therapeutic intervention in patients affected by type-2 diabetes mellitus [190] and in a weight management clinical study [191].

Anti-inflammatory and positive modulation of the immune system are the main health claims regarding probiotic properties. *Lpb. plantarum* IS-10506, from *danhi*, was successfully administered as a dietary early intervention to stimulate humoral and intestinal immune response in healthy preschool children [192,193], as well as to treat children affected by atopic dermatitis, a chronic recurrent inflammatory skin disease characterized by an immunity dysregulation [194]. The capacity to ameliorate quality of life in patients affected by atopic dermatitis via inflammation reduction and the immunomodulatory effects (i.e., IgE attenuation, reduction in eosinophil count) of *Lpb. plantarum* CJLP133 and *Lpb. plantarum* YIT 0132, isolated from kimchi and other fermented foods has been also shown [195,196]. *Lpb. plantarum* YIT 0132, administrated in fruit juice, has also been applied in the treatment of allergic syndromes which are widespread in Japan, such as perennial allergic rhinitis and Japanese Cedar Pollinosis, both of which are characterized by acute and sometimes severe inflammation status [196,198].

Amelioration of oxidative stress and inflammation by different food-associated *Lpb. plantarum* strains, (*Lpb. plantarum* P8 and PS128) has been observed in an emerging research field aiming to investigate the role of probiotics in exercise physiology and endurance performance [199,200,201,203,204]. Huang and coworkers reported a clear alleviation of exercise-induced inflammation with enhanced exercise performance in triathletes, suggesting a potential ergogenic role of *Lpb. plantarum* PS128 in high intensity training lifestyles [200].

Finally, with emergent health claims based on the gut–brain axis, *Lpb. plantarum* strains from food origin have proven to be promising probiotic candidates with beneficial effects on brain health. The dairy isolate, *Lpb. plantarum* DR7, has been shown to be efficient in mental stress conditions by reducing anxiety and stress, improving cognitive functions via stimulation of dopamine and serotonin pathways [208,209]. Improvements of cognitive function with increased serum brain-derived neurotrophic factor were also observed after 12-week administration of *Lpb. plantarum* C29 (kimchi isolate) in combination with fermented soybean in a clinical study enrolling 100 adults with mild cognitive impairments [211]. Interestingly, a beneficial effect of *Lpb. plantarum* PS128 as a dietary intervention in children with autism spectrum disorder was recently reported [202].

Diverse food-associated *Lpb. plantarum* strains have proven to be a naturally safe and efficient strategy for disease prevention in healthy subjects, as well as suitable interventions for various pathological conditions, as already demonstrated by human strains of the same species, such as the well-documented *Lpb. plantarum* 299v and *Lpb. plantarum* TENSIA. For a number of these strains from food origin, beneficial effects have been documented with in vitro and in vivo studies, as well as human trials. However, doses, time of treatment, and, often, molecular mechanisms, have not yet been defined. Ongoing and future studies should evaluate the effectiveness of each probiotic, in terms of adequate doses and treatment time, for the amelioration of specific diseases, taking into account the strain specificity and not overlooking the understanding of the mode of action of the probiotic at a molecular level.

## 6. Conclusions

In this review, we summarized the latest in vitro, in vivo and clinical evidence for the health-promoting properties of food-borne *Lpb. plantarum* strains. As mentioned, such strains can have a positive impact on host health by exerting immunomodulatory, antioxidant and antigenotoxic properties, among others. As a result, the origin of microorganisms is becoming less of a criterion for probiotic selection, and new evidence points to fermented foods as a rich source of live and active bacteria. In particular, strains belonging to the species *Lpb. plantarum*, that can be found in different fermented foods (sourdhough, table olives, cheeses), have shown to exert in vitro anti-inflammatory and antioxidative properties similar to those isolated from the GI tract [121,122]. Additionally, bacteria directly isolated from fermented foods may have advantages in food making processes due to their long history of adaptation to fermentation environments, thereby overcoming technological obstacles associated with the use of difficult-to-handle probiotic bacteria.

However, the inherent variability amongst strains and the lack of conclusive and reproducible results is creating conflicts for industry and scientific partners, with food-borne strains falling into legislative voids. Moreover, in most cases, the precise mechanisms by which food-associated strains exert their mechanisms of action are not well elucidated. As proposed by other authors, the complexity of these mechanisms gives rise to incomprehension about whether these benefits are the result of a direct effect mediated by the cell surface or by the secondary effects of metabolites produced under a given set of forming conditions. This difficulty is exacerbated when the GI ecosystem comes into play. Thus, further in vitro, in vivo and clinical trials must to be performed in order to elucidate the nature of these complex networks. In order to solve these problems, the scientific community has proposed to move towards a precision probiotic tactic, designed according to target-based discovery strategies and person-centric trials. This strategy will deepen our understanding of the mechanistic activity and host response, which will help in the design of probiotics and functional microbes for specific therapeutic purposes [212].

## Figures and Tables

**Table 1:** Most characterized plantaricins produced by *Lpb. plantarum* strains isolated from various fermented foods.

Strain Names	Isolation Source	Plantaricins Names	Sensitive Microbes	Reference
*Lpb. plantarum* ST28MS	Molasses	Plantaricin ST28 MS	*Lacticaseibacillus casei*, *Staphylococcus aureus*, *Enterococcus faecalis*, *Pseudomonas aeruginosa*, *Escherichia coli* and *Acinetobacter baumanii*	[45]
*Lpb. plantarum* LMG 2379	Wine	Plantaricin W	Gram-positive bacteria including *S. aureus*, *Listeria innocua* and *E. faecalis*	[50]
*Lpb. plantarum* C19	Fermented cucumber	Plantaricin C19	*Listeria grayi*	[51]
*Lpb. plantarum* 423	Sorghum beer	Plantaricin 423	*Bacillus cereus*, *Clostridium sporogenes*, *E. faecalis*, *Listeria* spp. and *Staphylococcus* spp.	[52]
*Lpb. plantarum* ST8SH	Salami	Plantaricin ST8 SH	*Enterococcus* spp., *Lactobacillus* spp., *Listeria* spp., *Streptococcus* spp. and *Klebsiella pneumonia*	[53]
*Lpb. plantarum* UG1	Dry sausage	Plantaricin UG1	*Listeria monocytogenes*	[55]
*Lpb. plantarum* LTF 154	Fermented sausage	Plantaricin 154	*E. faecalis*, *Bacillus* spp., *Staphylococcus* spp. and *Salmonella enterica* serovar Typhimurium	[56]
*Lpb. plantarum* SA6	Fermented sausage	Plantaricin SA6	*Lpb. plantarum*, *Levilactobacillus brevis*, *Leuconostoc* spp. and *L. grayi*	[57]
*Lpb. plantarum* TMW 1.25	Sausage fermentation	Plantaricin 1.25L	*Lactobacillus* spp.	[58]
*Lpb. plantarum* BF001	Spoiled catfish fillets	Plantaricin F	*S. aureus*, *S.* Typhimurium, *L. monocytogenes*, and *P. aeruginosa*	[59]
*Lpb. plantarum* LL441	Cabrales cheese	Plantaricin C	Gram-positive bacteria including *Bacillus subtilis*, *E. faecalis*, *C. sporogenes,* and *Clostridium tyrobutyricum*	[60,61]
*Lpb. plantarum* LC74	Crude goat’s milk	Plantaricin LC74	*Lpb. plantarum*, *Lev. brevis*, *Lentilactobacillus buchneri*, *Leuc. paramesenteroides*, *Bacillus stearothermophilus*	[62]
*Lpb. plantarum* K25	Kimchi	Plantaricin K25	*B. cereus* and *L. monocytogenes*	[63]
*Lpb. plantarum* ST31	Sourdough	Plantaricin ST31	*Lactobacillus* spp., *Leuconostoc* spp., *Pediococcus* spp., *Streptococcus* spp., *Bacillus* spp. and *S. aureus*	[64]
*Lpb. plantarum* 510	Koshu vineyard	Plantaricin Y	*L. monocytogenes*, *Weissella* spp., *Lactococcus lactis*, *Streptococcus salivarius* and *B. subtilis*	[65]
*Lpb. plantarum* LPCO10	Green olives fermentations	Plantaricin S Plantaricin T	*Clostridium* spp., *Propionibacterium* spp, *E. faecalis*	[66]
*Lpb. plantarum* 163	Fermented vegetables	Plantaricin 163	*S. aureus*, *L. monocytogenes*, *Bacillus pumilus*, *B. cereus*, *Micrococcus luteus*, *Streptococcus thermophilus*, *Lacticaseibacillus rhamnosus*, *E. coli*, *P. aeruginosa* and *Pseudomonas fluorescens*	[67]
*Lpb. plantarum* JLA-9	Fermented cabbage	Plantaricin JLA-9	*Bacillus* spp., *Clostridium* spp., *S. aureus*, *M. luteus*, *P. fluorescens*, *Serratia marcescens*, *E. coli*, *Salmonella* spp. and *Proteus mirabilis*	[68]
*Lpb. plantarum* C-11	Fermented cucumber	Plantaricin A Plantaricin EF Plantaricin JK	LAB species such as *Lactobacillus* spp., *Pediococcus* spp., *Leuconostoc* spp. and *Streptococcus* spp.	[69,70]
*Lpb. plantarum* ZJ008	Fresh milk	Plantaricin ZJ008	*Streptomyces citreus, M. luteus*, *S. aureus*, *E. coli*, *Shigella flexneri*, *Vibrio parahaemolyticus*, *L. monocytogenes* and *P. aeruginosa*	[71]
*Lpb. plantarum* ZJ5	Fermented mustard	Plantaricin ZJ5	*S. aureus*, *L. monocytogenes*, *S. flexneri*, *P. aeruginosa*, *Shigella dysenteriae*, *E. coli* and *Salmonella* spp.	[72]
*Lpb. plantarum* NRIC 149	Pineapple	Plantaricin 149	*Enterococcus hirae*, *Pediococcus acidilactici*, *Pediococcus cerevisiae*, *Lactobacillus* spp.	[73]
*Lpb. plantarum* BFE 905	Ready-to-eat salad	Plantaricin D	*Latilactobacillus sakei* and *L. monocytogenes*	[74]
*Lpb. plantarum* OL15	Algerian fermented olives	Plantaricin OL15	*Lactobacillus* spp.*, Lactococcus* spp. and *Propionibacterium* spp.	[75]
*Lpb. plantarum* DL3	Chinese pickled cabbage	Plantaricin DL3	*P. aeruginosa, P. fluorescens, Shewanella putrefaciens, Psychrobacter spp.*, *L. monocytogenes*, *B. cereus*, *Bacillus licheniformis*	[76]
*Lpb. plantarum* Q7	Yak yogurt	Plantaricin Q7	*L. monocytogenes, S. aureus, E. coli, P. fluorescens, P. putida, P. aeruginosa, Shigella flexneri, Shigella sonnei, S.* Typhimurium	[77]
*Lpb. plantarum* KLDS1	Chinese fermented cream	Plantaricin MG	*L. monocytogenes*, *S. aureus*, *S.* Typhimurium and *E. coli*	[78]
*Lpb. plantarum* LPL-1	Fermented fish	Plantaricin LPL-1	*L. monocytogenes, S. aureus, Bacillus amyloliquefaciens, B. pumilus, E. faecalis, Lactobacillus* spp., *Lact. lactis*	[79,80,81]
*Lpb. plantarum* GZ1-27	Traditional kipper	Plantaricin GZ1-27	*Brochothrix thermosphacta, P. fluorescens, A. baumannii, B. cereus, S. aureus, S.* Typhimurium*, L. monocytogenes and E. coli.*	[82]
*Lpb. plantarum* SLG1	Yak cheese	Plantaricin SLG1	*B. subtilis, B. cereus, Bacillus megaterium, M. luteus, B. thermosphacta, Clostridium butyricum, S. aureus, L. innocua, L. monocytogenes, E. coli, P. aeruginosa, Enterobacter cloacae, Salmonella paratyphi, Saccharomyces cerevisiae and Candida albicans*	[83]
*Lpb. plantarum* BM1	Fermented Chinese meat product	Plantaricin BM1	*E. faecalis, L. monocytogenes, Lpb. pentosus*, *Lpb. plantarum*, *Shigelladysenteriae, E. coli, S. aureus, S.* Enteritidis	[84]
*Lpb. plantarum* MBSa4	Brazilian salami	Plantaricin MBSa4	*S. aureus, L. innocua, Listeria welshimeri, L. monocytogenes, E. hirae, E. faecium, Limosilactobacillus fermentum, Lat. sakei, Penicillium roqueforti, Penicillium expansum, Fusarium* sp., *Mucor plumbeus*, *Cladosporium* sp., *Debariomyces hansenii*	[85]

**Table 2:** Clinical studies of food-associated *Lpb. plantarum* strains showing efficacy for treatment of several disorders.

Bacteria	Origin	Dose	Health Condition	Subjects and Timeline	Main Impact	Main Outcomes	Reference
*L. curvatus* HY7601 *Lpb. plantarum* KY1032	Korean traditional fermented cabbage	5 × 10^9^ CFU/d in powder	Hypertriglyceridemia	92 adults 12 weeks	Cholesterol-lowering effect	Triglyceride-lowering effects through reductions in plasma metabolites, fatty acid primary amides and lysoPC	[185]
		0.5 × 10^10^ CFU/d in powder	Hypertriglyceridemia	128 adults 12 weeks	Cholesterol-lowering effect	reduction of triglycerides and increase of apo A-V and LDL particle size	[186]
		5 × 10^9^ CFU/d in powder	Overweight subjects	120 adults 12 weeks	Weight loss	Reductions in body weight, body fat percentage and body fat mass	[187]
		5 × 10^9^ CFU/d in powder	Overweight subjects	66 adults 12 weeks	Weight loss	Weight loss and adiposity reduction associated with an increase in medium-chain acylcarnitines	[188]
*Lpb. plantarum* ECGC 13110402	Dairy isolate	4 × 10^9^ CFU/day in capsules	Hypercholesterolaemia	49 adults 12 weeks	Cholesterol-lowering effect	Reduction in LDL and Total cholesterol, reduction of triacylgycerides and increase of HDL in over 60′s	[189]
UB0316 *containing Lpb. plantarum UBLP-40*	Fermented foods	5 × 10^9^ CFU/day in capsules	Type 2 diabetes mellitus	79 adults 12 weeks	Weight loss	Weight loss, improvements in glycemic control via reduction in HbA1c levels	[190]
		5 × 10^9^ CFU/day in capsules	Overweight/obesity conditions	71 adults 12 weeks	Weight loss	Reduction of BMI, body weight and WHR in overweight/obese adults	[191]
		2 × 10^11^ CFU/day Microencapsulated in powder	Healthy preschool children	48 one-two years old children 90 days	Immunomodulation	Increased humoral immune response, as well as improved zinc status	[192]
*Lpb. plantarum* IS-10506	*dadih* Indonesian traditional fermented buffalo milk	2.3 × 10^10^ CFU/day Microencapsulated in powder	Immature intestinal immune system	38 one-two years old children 90 days	Immunomodulation	Stimulation of TGF-β1, increased sIgA production, with a significant correlation between TGF-β1/TNF-α and fecal sIgA	[193]
		10^10^ CFU/day Microencapsulated in powder	Atopic dermatitis	22 children 12 weeks	Immunomodulation Anti-inflammatory activity	SCORAD and levels of IL-4, IFN-γ, and IL-17 were significantly lower decrease in clinical Symptoms through down regulation of Th2 adaptive immune response	[194]
*Lpb.plantarum* CJLP133	Kimchi	10^10^ CFU/day powder in airtight alu-bags	Atopic dermatitis	83 children 16 weeks	Immunomodulation Anti-inflammatory activity	Reduced SCORAD score, eosinophil count and cytokines levels (IL-4 and IFN-γ)	[195]
		8 × 10^10^ cells/day in fermented citrus juice	Perennial allergic rhinitis	33 adults 8 weeks	Immunomodulation with antillaergic effects	Reduction of nasal symptoms. attenuation of Th2 cells, total IgE and ECP	[196]
*Lpb.* *plantarum YIT 0132*	Fermented foods	8 × 10^10^ cells/day in fermented citrus juice	Atopic dermatitis	32 + 18 adults 8 + 8 weeks	Immunomodulation with antillaergic effects	Reduced symptoms with immunomodulatory effect via attenuation of IgE and ECP	[197]
		8 × 10^10^ cells/day in fermented citrus juice	Japanese Cedar Pollinosis	42 adults 8 weeks	Antiallergic effects	Reduction of allergy symptoms	[198]
		3 ×10^10^ CFU/day in capsules	Triathlete’s microbiota	20 Triathletes 4 weeks	Endurance performance amelioration via gut microbiota	GI health and physiological homeostasis maintenancenduring endurance exercise through functional microbiota modulation	[199]
*Lpb. plantarum PS128*	*Fu-tsai* Taiwan fermented mustard greens	3 × 10^10^ CFU/day in capsules	Exercise-induced inflammation	N.R.	Antioxidative and anti-inflammatory activities	Oxidative stress and inflammation alleviation	[200]
		3 × 10^10^ CFU/day in capsules	Exercise-Induced oxidative stress and inflammation	18 Triathletes 8 weeks	Antioxidative and anti-inflammatory activities	Oxidative stress alleviation, decreased pro-inflammatory parameters, enhanced exercise performance	[201]
		3 × 10^10^ CFU/day in capsules	Autism spectrum disorder	80 children 4 weeks	Mental health	Age-dependent amelioration of autism symptoms	[202]
*Lpb. plantarum P8*	Traditionally Mongolian fermented sour milk	6 × 10^10^ CFU/day in tablets	Healthy adults	33 adults 4 weeks	Gut microbiota and Immunomodulation	Time- and age- related changes in fecal sIgA, TBAs, and SCFAs levels Beneficial alteration of gut microbiota	[203]
		2 × 10^10^ CFU/day powder in sachet	Mental stress conditions	103 adults 12 weeks	Antistress and anti-inflammatory activities	Alleviation of selected stress, anxiety, memory and cognitive symptoms in stressed adults with reduction of pro-inflammatory markers and enhanced memory and cognitive traits	[204]
*Lpb. plantarum TWK10*	Taiwan pickled cabbage	3 × 10^10^ CFU/day 9 × 10^10^ CFU/day in capsules	Exercise physiology	54 adults 6 weeks	Endurance performance amelioration	Enhanced exercise performance in a dose-dependent manner correlated with better physiological adaptation (body fat significantly decreased and muscle mass significantly increased)	[205]
		1 × 10^11^ CFU/day in capsules	Endurance performance	16 adults 6 weeks	Endurance performance amelioration	Significantly higher endurance performance and glucose content	[206]
*Lpb. plantarum* DR7	Cow milk	10^9^ CFU/day powder in aluminium sachet	Upper respiratory tract infections	109 adults 12 weeks	Immunomodulation with anti-inflammatory activities	Improvements of nasal symptoms and URTI frequency by improving inflammatory parameters and enhancing immunomodulatory properties	[207]
		10^9^ CFU/day powder in aluminium sachet	Mental stress conditions	124 adults 12 weeks	Antistress and anti-inflammatory activities	Modulation of stress-induced bowel movement and gut microbiota in association with dopamine and serotonin pathways	[208]
		10^9^ CFU/day powder in aluminium sachet	Mental stress conditions	111 adults 12 weeks	Antistress and anti-inflammatory activities	Reduced plasma cortisol and pro-inflammatory cytokines. Reduction of stress and anxiety, improved cognitive and memory functions with stimulation of serotonin and dopamine-norepinephrine pathway	[209]
*Lpb. plantarum* SN13T	Plant-derived	2 × 10^8^ CFU/g in yogurt	Constipation	68 adults 6 weeks	Gut functions improvement	Constipation restoring effects with improved serum lipid contents and liver functionality	[210]

* Abbreviation list. lysoPC: lysophosphatidyl choline; apo A-V: apolipoprotein A-V; LDL: low density lipoprotein; SCORAD: Scoring Atopic Dermatitis Index; IL-4: interleukin 4; IFN-γ: interferon- γ; IL-17: interleukin 17; Th2: T helper type 2; TGF-β1: Transforming growth factor-β1; sIgA: secretory immunoglobulin A; TNF-α: Tumor necrosis factor- α; URTI: Upper Respiratory Tract Infections; HDL: High density lipoprotein; IgE: immunoglobulin E; ECP: eosinophil cationic protein; TBAs: total bile acids; SCFAs: shortchain fatty acids; BMI: body mass index; WHR: waist-to-hip ratio; hs-CRP: high-sensitivity C-reactive protein; N.R.: not reported.

## Data Availability

Not applicable.
